# Multi-Agent Interaction to Assist Visually-Impaired and Elderly People

**DOI:** 10.3390/ijerph19158945

**Published:** 2022-07-22

**Authors:** Juliana Damasio Oliveira, Debora C. Engelmann, Davi Kniest, Renata Vieira, Rafael H. Bordini

**Affiliations:** 1 School of Technology, Pontifical Catholic University of Rio Grande do Sul, Porto Alegre 90619-900, Brazil; debora.engelmann@edu.pucrs.br (D.C.E.); davi.silva01@edu.pucrs.br (D.K.); rafael.bordini@pucrs.br (R.H.B.); 2 Department of Informatics, Bioengineering, Robotics and Systems Engineering, University of Genoa, 16145 Genoa, Italy; 3 CIDEHUS, University of Évora, 7000-809 Évora, Portugal; renatav@uevora.pt

**Keywords:** multi-agent systems, daily living assistance, elderly care, visual impairment assistance

## Abstract

A voice-controlled smart home system based on conversational agents can address the specific needs of older people, proactively providing support, compensating for cognitive decline, and coping with solitude, among other features. In particular, Multi-Agent Systems (MAS) platforms provide considerable support for complex adaptive systems that are naturally distributed and situated in dynamic environments, such as Ambient intelligence (AmI) applications. Such autonomous intelligent agents are capable of independent reasoning and joint analysis of complex situations to support high-level interaction with humans, besides providing typical characteristics of MAS, such as cooperation and coordinated action. In this context, we developed an approach using a MAS previously evaluated for visually impaired users, where most of the system’s functionalities are also helpful for the elderly. Our methodology is based on the four steps of the interactive design process. As a result, we determined that our approach has elements that allow for natural interaction with users, and we identified and discussed improvements and new features for future work. We believe that our findings can point to directions for building AmI systems that are capable of more natural interaction with users.

## 1. Introduction

In recent years, there has been an increase in life expectancy, mainly due to technological advances. According to the WHO (World Health Organisation), the number of older people globally is expected to increase to 1 billion by 2030. Moreover, by the year 2050, people over 60 may comprise more than 25% of the population [1]. For instance, in Brazil, the estimate increased from 45.5 years in 1940 to 75.8 years in 2016 [2]. Nowadays, he increase in life expectancy, and the consequent ageing of the population in general, represent significant challenges for modern societies [3]. One of the challenges is that with old age comes chronic health problems and sensory and cognitive impairments. As a result, many seniors end up with vision (low vision and blindness) and memory problems. In addition, families have less and less time to care for their elderly relatives, and their isolation is a genuine concern these days. This situation is exacerbated when these older adults live alone [3].

As society is ageing, it is necessary to provide tools so that people can live more safely and for longer in their own homes. The development of technologies for ambient assisted living (AAL), which is one of the most promising areas of ambient intelligence (AmI), is an excellent opportunity to help users with special needs, such as those who live with disabilities, illness, or ageing [4], in order to promote the possibility of living alone longer at home [5]. In addition, AAL can be used to monitor, assist, and promote a healthy environment [6] A voice-controlled smart home would answer the specific needs of elderly and disabled people [7], such as conversational agents, chatbots, and virtual assistants.

The complexity of AmI requires advanced control systems [8]. Multi-Agent Systems (MAS) are considered suitable tools for complex adaptive systems, especially for those that are distributed and dynamic, such as AmI [9,10]. Furthermore, the use of intelligent agents provides analysing information on distributed sensors [11,12,13]. These agents are capable of both independent reasoning and joint analysis of complex situations in order to achieve a high level of interaction with humans [14], enjoying all the characteristics of MAS systems of cooperation, organisation, and intelligence [13].

In Human-Computer Interactions (HCIs), the ambient intelligence presents the challenge of removing desktops and embedding computers in the environment, so it becomes imperceptible to humans while surrounding them everywhere [15]. The involvement of HCIs in user interface design has an increasingly significant impact on building a practical ambient intelligence such as a smart home [16]. In addition, people with disabilities, especially those who are visually impaired, could enjoy ambient intelligence if the interfaces were accessible and usable, allowing easy and effective house control [17].

In this paper, we discuss a multi-agent architecture used as a basis for an AAL system that was initially developed and evaluated with visually impaired users, where most of the system’s functionalities are also helpful for older people. We also present an evaluation with HCI experts to assess how well such a system would support elderly users in finding objects and controlling aspects of their environment.

## 2. Related Work

This section details the specific publications that use multi-agent systems for ambient intelligence. Most of the publications are for elderly and disabled people [8,18,19,20,21,22].

Mostafa et al. [21] presented an architecture of Adjustable-Autonomous Multi-agent IoT (AAMA-IoT) system which aimed to resolve some of the IoT management of control and application interface challenges. This is the system which is applied in an elderly smart home simulation. The architecture consists of a physical layer for perception and interaction, a network layer for data transport and system communication, and an application layer. It considers that agents can have different levels of autonomy related to their interaction behaviours. The authors divide them into five modes, from Not-Active (no sensing and no actions) to Proactive (sensing and proactive actions), and modes that can be adjusted given the agent’s nature. They implemented the simulation in Java and Java Agent Development (JADE) framework and used a dataset that consists of real-world scenarios of an IoT-based smart home with instances of 14 things (devices/sensors) recorded for 28 days. It reached an accuracy of 96.97%, with agents determining the interaction between the things based on their corresponding tasks and elderly daily activities.

Sernani et al. [18] proposed a multi-agent system called “Virtual Care” to help an elderly or disabled patient to monitor health conditions and control the environment. Virtual Care is a system for managing a network of distributed sensors composed of environmental and biometric sensors while being an interface layer between the network, the person assisted, their relatives, and the medical team. In addition, it includes an objective-oriented reasoning module, i.e., the Virtual Care agent, based on the BDI architecture. Sensors and actuators are implemented using the JADE framework, while the BDI agent representing the Virtual Care agent and the Registration Agent are implemented using the JASON language. This architecture provides user interaction through audio and gestures but is not currently used. They evaluated the system using simulations.

Fraile et al. [8,19] created HoCaMa [19] and AMADE [8], a hybrid multi-agent architecture that facilitates remote monitoring and care services for disabled patients, for example, alerts and warnings, such as remembering to take medications, control, and supervision home care environment. The architecture combines deliberative and reactive agents and incorporates Java cards, RFID, and SMS. In the implementation, FIPA ACL and SOAP protocols were used. HoCaMa/AMADE were reasoning and planning mechanisms: case-based reasoning and case-based planning actions and plans are selected according to their utility functions. The architecture was implemented in one home of five patients and was tested for 30 days. They compared HoCaMa/AMADE with ALZ-MAS (another architecture created by the same authors). The case study consisted of analysing the functioning of both architectures in a test environment. The system sends alerts to the cell phone, but the user can not interact directly with the system to make requests. Unfortunately, this architecture is no longer available for download because the authors have sold it to a private company.

Fiol-Roig et al. [20] developed a virtual agent for disabled and elderly people assistance, called Intelligent Butler. The agent performs health care, control devices, and house security; informs about the goods available and those exhausted in the house, such as food or medication; and preferences and schedule. The system incorporates sensors and domotic devices. The agent uses perception-action vectors and object attribute tables. This system was evaluated with a virtual simulation of a home domotics environment. The person assisting does not interact with the system.

Martínez et al. [22] presented a device designed to assist elderly people in their homes, which in concept is a multi-agent system, meaning it can be adapted to work in smart homes with IoT. The main idea is to create a desk that can maintain a record and manage the administration of the user’s medications, besides including three other agents with sensors to monitor the interaction with the assistant. Each agent receives a level of autonomy, which defines the degree of interaction that the agents are expected to maintain with the users. This includes: autonomous agents, which have rare interactions and prioritise user safety (agent: gas leak/fire alarm); semi-autonomous agents, which should interact regularly and prioritise the user’s health (agents: light control and pill dispenser); and non-autonomous agents, with which the user should often interact, and which have comfort as a priority (agent: fan). The authors simulate the use of the device by considering different probabilities of user intention and agent reaction. They conclude that the device itself represents the early stages of development of a system, and that the system is performing as expected, with space for further work.

Our approach differs from those presented in this section because the presented architectures did not add forms of interaction in which the users could make requests to the system. In addition, the significant differential in our work is the use of chatbots and the use of the Portuguese language.

## 3. Method

In this section, we present the methodology that are based on interaction design method [23]. We organised our research in the following activities.

**Establish requirements**: we conducted *Investigations on end-users’ needs*, performed by conducting 3 studies:
  - Survey with end-users: developed an online survey that was answered by PVI, 27 participants answered it [24].
**Design alternatives**:
  - Mapping study: we performed a mapping study to identify the techniques, technologies, architectures, methodologies, features, and evaluations most used or conducted in the literature [25].
  - Then, we performed a systematic literature review to identify *Guidelines to improve user interaction with ambient intelligence systems* [26].
  - We created and evaluated our first design idea that included the main feature pointed out by the end-users. We evaluated with an end-user. This evaluation provided insights to guide our next steps.
**Prototypes**: we created an approach for Ambient Assisted Living using a multi-agent system. This approach takes into consideration the established requirements.
**Evaluate**: Our approach was evaluated by seven HCI specialists and seven end-users [27]. In the current work we evaluated the approach with HCI specialists to verify its application to another end-users, elderly people.

We analysed all the data collected using the quali–quanti approach.

## 4. An Approach to Ambient Assisted Living

This section presents our MAS approach, which was initially developed to assist visually impaired people in their homes. However, most of the system’s features are also helpful for older people. We chose to create a multi-agent system because, in general, it is designed to handle environments containing distributed and dynamic resources [9,10]. In addition, using this system, it is possible to add more features by only adding more capabilities to the agents, more agents, and artefacts.

**Figure 1:**
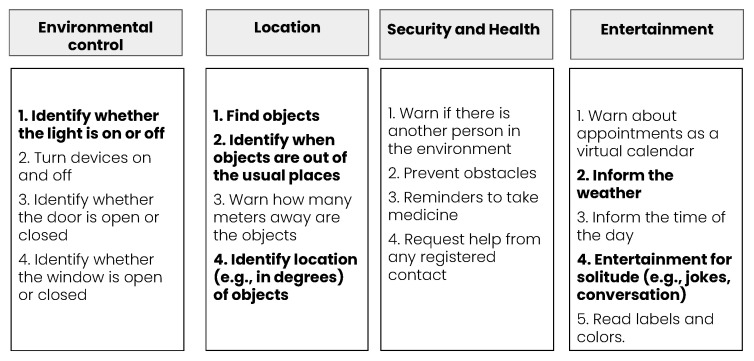
Features categories—the ones that are highlighted we implement.

In our previous research [24,25], we identify the main features that visually impaired people need in their homes. Then, we grouped these features into several categories, depicted in Figure 1, and include the highlighted features in our approach. The categories are as follows:

**Environmental control:** This category has features related to the environment, such as identifying whether the light is on or off; identifying whether the window is open or closed; identifying whether the door is open or closed; and turning devices on and off.
**Location:** This category shows the features related to the objects’ location, such as: finding objects; identifying when objects are out of the usual places; identifying location (e.g., in degrees) of objects; and warning the user of how many meters away the objects are.
**Security and health:** This category brings the features related to the safety and health of the person assisted, such as: warning the user if there is another person in the same environment; requesting help from any registered contact; detecting obstacles; and reminders to take medicine.
**Entertainment:** This category encompasses features related to the entertainment for the assisted person, such as: informing the current time of the day; informing about the weather; warning about appointments as a virtual calendar; entertainment for solitude (e.g., jokes, conversation); and reading labels and colours.

Furthermore, during the design of the system, we selected some guidelines introduced in our previous research [26], as described below:

- 1. Usability and Accessibility: We primarily consider ease of use, understanding, and learning.
- 2. Natural Interaction: We use a conversational interface to provide a natural interaction for users. We are also concerned with inserting natural and intuitive commands to perform tasks, including terms and intuitive feedback.
- 3. Multimodality: the user can exchange information via voice or text with the system.
- 6. Effectiveness and Efficiency: the user needs a few steps during a dialogue with the system to perform a task. The user does not need to log into the system for each use. While this can be a threat to the user’s security, it is an enabler for the user. Furthermore, considering that the user needs to log in to their smartphone, this risk is minimised. The system has effective communication.
- 7. Instructions, Suggestions, and Support: We have inserted a help command in the system with the system’s main features that can be requested at any time. When the user requests “Help”, the system informs which features the user can request.

### 4.1. Approach Overview

**Figure 2:**
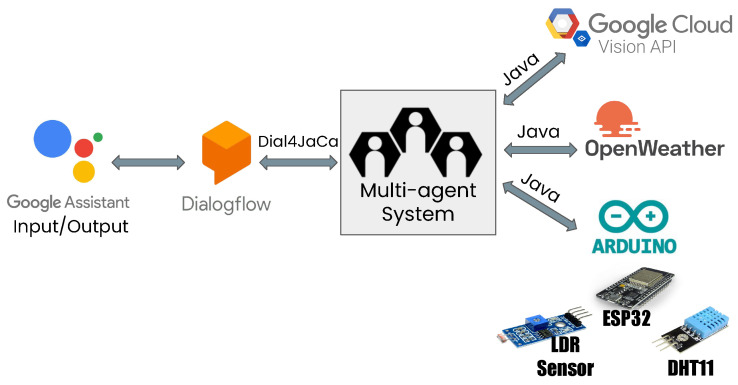
High-level view of our approach.

Figure 2 shows a high-level view of how our approach works. We name our approach Homer. First, the user interacts with the system through voice commands (according to user preference) using the Google assistant. The Google assistant is integrated with the Dialogflow platform, where we created our conversational agent. Our agent speaks in Portuguese. We chose DialogFlow because it offers several integrations and is available for free. DialogFlow sends action requests to our multi-agent system, which performs the requested actions on it based on the current state of the environment. Our multi-agent system also integrates with Google cloud vision API (https://cloud.google.com/vision, accessed on 16 February 2022 ), Open Weather API (https://openweathermap.org/api, accessed on 16 February 2022), and through Arduino (https://www.arduino.cc/en/software, accessed on 16 February 2022) we have communication with peripheral, sensors and actuators such as light and humidity sensors. For those peripherals, we specifically used an ESP32 micro-controller configured with the Arduino IDE to communicate with the system through HTTP communication and the two sensors through wires. The sensors used were an LDR light sensor module that uses the LDR component to measure the light incidence and the DHT11 temperature and humidity sensor, which has high reliability since it is calibrated before distribution. Thus, depending on the user’s request, Homer calls one of the available integrations and then updates the user about the system’s current state using voice feedback.

The multi-agent system was developed using the JaCaMo platform. It includes Jason, which is an interpreter that performs better than other agent programming languages [28]. In addition, using an organisation of agents (Moise) and environment (CArtAgO) is interesting in this scenario because Moise enables agents to perceive the roles available in the organisation, and CArtAgO enables us to model the devices in the environment.

### 4.2. The Multi-Agent System: Homer

**Figure 3:**
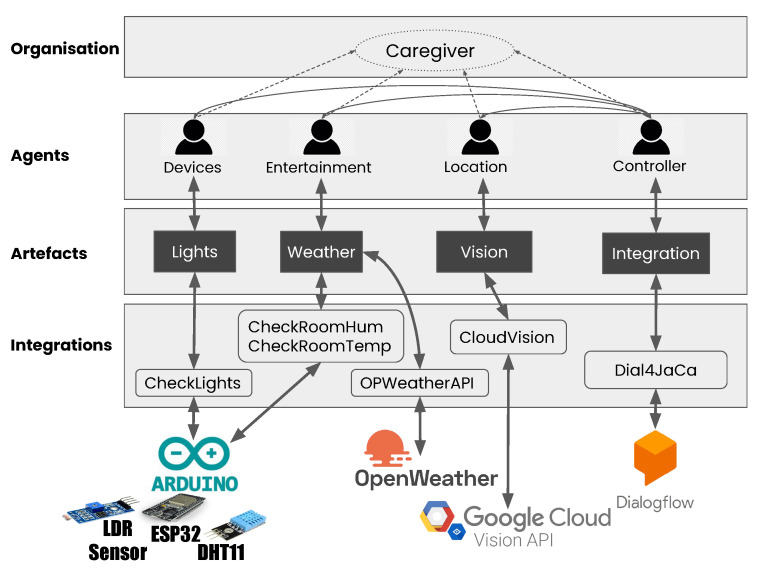
Multi-agent system structure.

**Figure 4:**
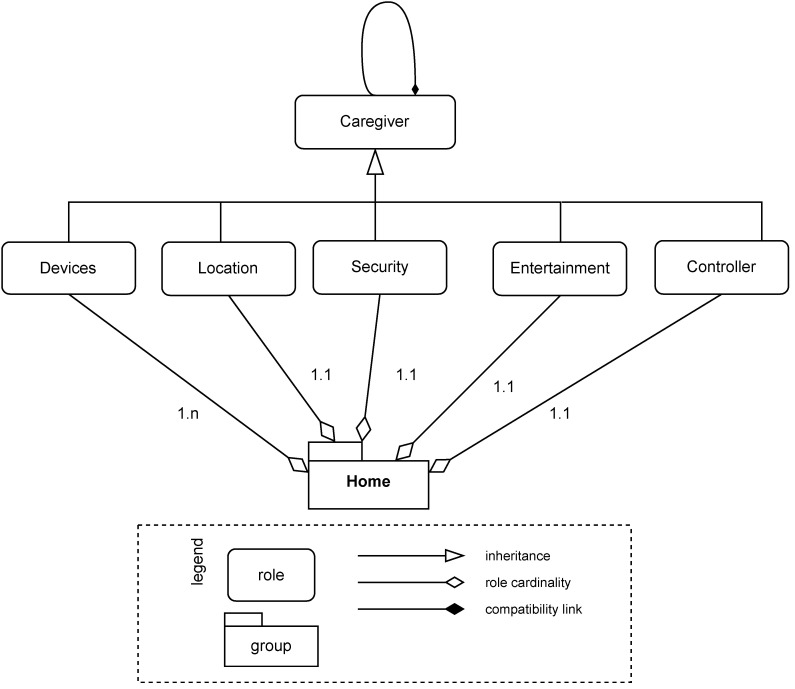
Multi-agent organisation.

The main elements of our multi-agent system (the implementation is available on Github https://github.com/smart-pucrs/Homer, accessed on 2 March 2022) are presented in Figure 3. It consists of four layers. The Organisation layer contains the main role of the Caregiver system. All agents extend this role, as we can see in the Agents layer. Figure 4 shows the roles and groups using Moise (in this proposal, we simplified Moise specifications to keep the focus on the main aspects.) notation [29]. Furthermore, in the Agents layer, we can see that a specific agent was created according to the categories of features. The Artefacts layer has all the artefacts in the system. The Integration layer has all the Java codes to access the APIs, which are then accessed by the Artefacts to communicate with the agents. This Integration layer allows us to easily change the technologies our multi-agent system communicates with, such as replacing Cloud Vision with YOLO or Dialogflow with another natural language understanding platform.

The information flow, that is, the user’s request, occurs as follows. Dialogflow sends the user’s request to our multi-agent system. To receive those requests, our system uses an integration between JaCaMo and Dialogflow called Dial4JaCa (https://github.com/smart-pucrs/Dial4JaCa, accessed on 2 March 2022) [30], which extends the open-source JaCaMo Rest project (https://github.com/jacamo-lang/jacamo-rest, accessed on 2 March 2022) [31]. It is represented by the Integration artefact, in Figure 3. Then, our Controller agent is responsible for forwarding the request depending on what it is about. For instance, when it is a request about an object location, a plan is triggered that forwards the request to our Location agent. Likewise, when the request is about the weather, another plan that forwards the request to the Entertainment agent is triggered. Similarly, when it comes to the status of a light or the temperature, the triggered plan forwards the request to the Device agent.

Each specific agent reacts according to the perceptions sent by the artefacts in our system. For instance, the Location agent consults the Vision Artefact, which then consults CloudVision, and the response is delivered to the Controller agent, which notifies the user.

We believe that with the structure we created, it is possible to be expanded by adding new features, and, in addition, it is also possible to change the integrations used in the system. For this, it would be enough to respect the data requested by the artefacts.

### 4.3. Implementation

This section will detail each agent’s responsibilities in our system.

#### 4.3.1. Controller Agent

The controller agent is responsible for all communication between DialogFlow and the specific agents; that is, it receives requests from the users and forwards them to the specific agents. Afterwards, it receives the agents’ conclusions and delivers them to Dialogflow.

#### 4.3.2. Location Agent

All *location features* were implemented using the Cloud Vision API. The Cloud Vision API needs images for object recognition. We have implemented a Java class that identifies and accesses the user’s webcam and takes pictures of the environment to provide those. This data is then processed by Cloud vision. It is important to mention that for each object, the Cloud Vision API returns the name of the object in English and the X and Y coordinates followed by a confidence degree. We translate the names of the objects to Portuguese using a service provided by Google cloud (https://cloud.google.com/translate, accessed on 22 February 2022). Moreover, we created a class named ObjectRepresentation in which Java methods calculate the degree position that each object is and whether it is on the right, left, or in front of the camera. All these calculations use the user’s webcam position as a reference. This information is accessed by the Vision artefact.

We implemented two ways to use the *find objects*. In the first one, the user can list all the objects in the image. This feature lists the number of objects and their respective names. In this feature, we chose not to present the locations of every object in the environment because the message could get too long and tedious for the user. In the second one, the user can search for a specific object and obtain its location, such as the degrees and whether it is on the right/left or in front of them.

Moreover, to search for a *specific object*, we provide additional information regarding the two closest objects related to the requested one. This is important in case the user cannot determine the precise location of the object given only the degrees and directions coordinates. We believe two objects provides a good balance in order to avoid overwhelming the user with information.

To determine those objects, the Location agent searches for the closest object on the X-axis of the image. Then, the second object is searched on the Y-axis of the image. Given a list containing all objects identified in an image, the agent iterates over it and selects the object that minimises the following equation:

$$diff=(x_{r}+x_{1})/2,$$

where $x_{r}$ is the x-coordinate of the requested object and $x_{1}$, similarly, is the x-coordinate of another object in the given list. The resulting object is then removed from the list. This process is repeated on the Y-axis to select the second object.

Next, we inform the requested object’s relative position (right or left) regarding the selected objects. To this end, the Location agent compares the objects’ x-coordinates. If $x_{1}$ is greater than $x_{r}$, the requested object is on the left of the other one. Conversely, if $x_{1}$ is less than $x_{r}$, the requested object is on the right.

**Figure 5:**
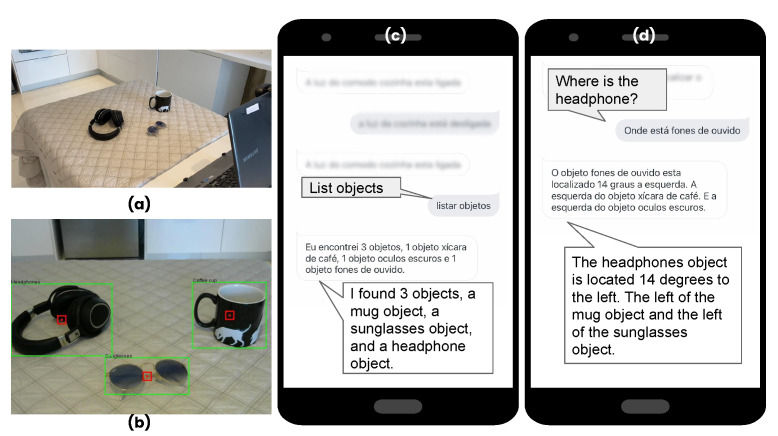
Homer execution (**a**) scenario, (**b**) scenario recognised by the camera, (**c**) find objects, and (**d**) find specific objects.

In some cases, there may be more than one object with the same name in the image (e.g., two books). In these cases, the system uses the first object it finds to return information. If there is only one closest object, the system lists in relation to that object only. If there is only the requested object and two more objects in the scene, the list of those objects is returned. Figure 5 shows the execution of the features of *find objects* and *find specific objects* with Homer.

**Figure 6:**
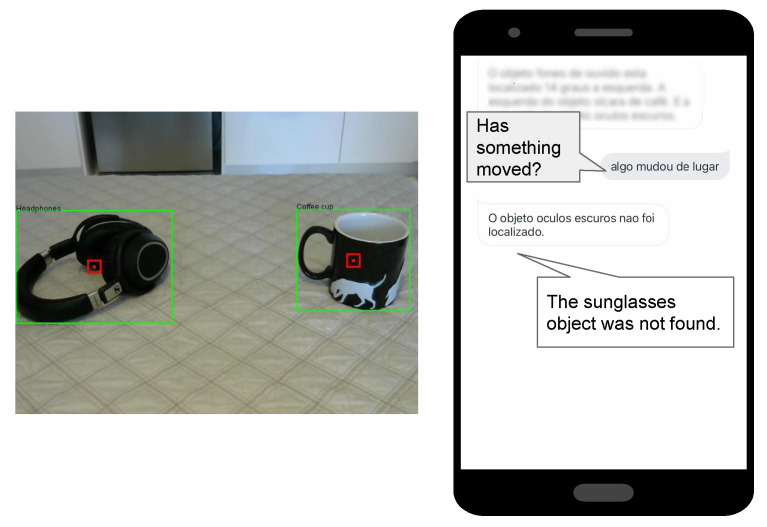
Homer execution—identify when objects are out of the usual places.

We created a feature to *Identify when objects are out of the usual places*. This feature allows the user to identify when an object has been moved away from its usual location or when new objects have appeared in the environment. We compare the image that was taken previously with a new picture. The Location agent is responsible for this task. The Location agent receives the string that comes from Vision Artefact that contains each object’s location in the last image, such as degrees and its relative direction. It has a plan to compare this information. If the new image contains the same data as the last image, it means everything is in the same place; if it is different, it means it has moved. If the object is not in the list of the old image, then it is added as a new object. Furthermore, if there is any object in the old image that was not found in the new one, then the Location agent informs that one object was not found. Figure 6 shows the execution of the *identify when objects are out of the usual places* feature. In that demonstration, we took the sunglasses object out of the scene.

#### 4.3.3. Devices Agent

**Figure 7:**
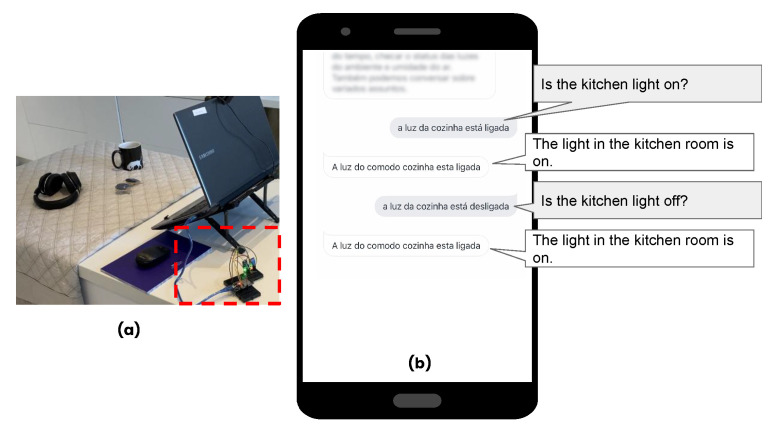
Homer execution (**a**) micro-controller board with sensors, and (**b**) identify whether the light is on or off.

We created a Devices Agent to control the environment; at the moment, it is responsible for *identifying whether the light is on or off* feature (see Figure 7). To do so, we used a micro-controller board to send data from a light sensor via Wifi, making it possible to check the status of the light (on/off). We access the board using the HTTP method.

#### 4.3.4. Entertainment Agent

**Figure 8:**
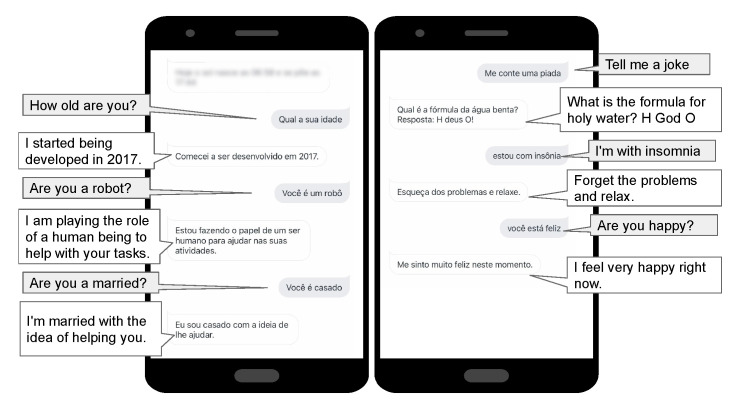
Homer execution—entertainment for solitude.

Our Entertainment agent is responsible for free conversation, telling jokes, and reporting the weather forecast. In the *Entertainment for solitude* feature. Dialogflow uses the concept of intent. Each intent has questions, e.g., “what the user says” and possible responses to the request. When the response is not fetched from an external service, they are registered directly in Dialogflow. A huge number of intents registered in Dialogflow makes the communication with the user more natural and diversified. With that in mind, we have entered 100 intents with the appropriate user-assignable questions and answers. It has intents that allow small talk, and that tell jokes to the user (See Figure 8). Additionally, we added a default fallback intent for when the agent does not recognise the user’s request. Thus, in this case, the user is informed “Sorry, but I do not understand.”

**Figure 9:**
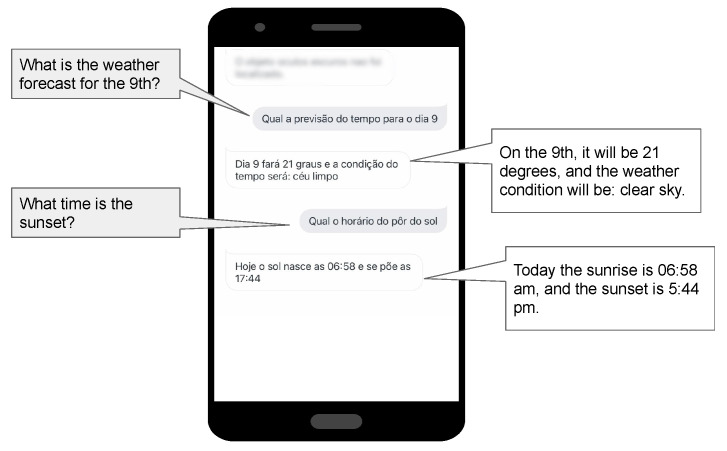
Homer execution—inform the weather.

The *Inform the weather* feature allows the user to determine the weather forecast for a specific day of the next seven days to come, or the current day (See Figure 9). The user can also check the sunset time and the humidity conditions for a particular day. Our Weather Artefact accesses the OPWeatherAPI class that uses the Open Weather API and makes those properties observable to the Entertainment agent.

## 5. Evaluation

We performed an evaluation with three HCI specialists. These specialists were intentionally selected according to their expertise in the HCI area. Our goal was to identify whether Homer’s existing features could also be used by older people, usability issues, and new features that serve these users.

### 5.1. Method

We asked specialists to watch a Homer demo video made available on YouTube (https://youtu.be/t8ZyKnPU32A, accessed on 25 February 2022). Then, we asked them to answer an online questionnaire. In that demo, there was a brief explanation about the whole system, our persona, and some scenarios for using the system. Afterwards, we run the scenarios using Homer. We performed the following features in the video: list objects, find a specific object, inform the weather, check if any object was out of place, help, and check lights.

In the online questionnaire, the main questions evaluated were: if the features presented would be helpful to older people. There was also an overall evaluation of the system with questions to identify whether the system is easy to use, easy to act on instructions, and easy to understand what actions are available. As well as, new features that they believe would be important to add to the system to assist older people in their homes. Furthermore, the positive and negative points of the system were asked. In this section, we analyse and discuss their answers.

Table 1 summarises the HCI specialists’ profile. As for the specialist profiles, they were between 29 and 50 years old, and included three males. S1 had experience with systems development for older people. He developed an application and websites for a retail store, which comprised the highest age group of customers and customers with little technological education. As for experience with virtual assistants, only one participant had never used one. Some virtual assistants cited were: Siri, Google Home, Google Assistant, and Alexa.

### 5.2. Results

The results indicated that all specialists strongly agree or agree that the Homer features are adequate for elderly people. Furthermore, the system is easy to use, easy to act on instructions, and easy to understand what actions are available. Concerning the “Which other features do you think would be important to add to the system to help elderly people in their homes?” question, S1 suggested adding a feature to remind the user to take medication, informing the time and medication. S2 suggested the possibility of controlling other smart appliances such as robot vacuum cleaners and washing machines, for instance. Finally, S3 suggested monitoring the user’s daily tasks and allowing contacting other people in case of emergency.

About the “What are the positive points of the system?” question, the users answered:

- “The main positive point is the ease of use since the system understands what the user says well.”(S1)
- “Thinking about the elderly, voice interaction is very positive, and so is the issue of identifying "lost" objects.” (S2)
- “help with daily task.”(S3)

Referring to the “What are the negative points of the system?” question, the users answered:

- “I don’t know if it’s a negative point, but it generated a doubt. How do you intend to locate objects throughout the house? Will multiple cameras integrated with the system be required?”(S1). Answering this question brought by the S1, today, the system has a single camera, but as we use multi-agent systems, more cameras and other devices can be added.
- “Not a negative, but all of a sudden, as an upgrade to the assistant’s voice, it would be interesting, so it has a less robotic and more natural voice.” (S2)
- “None.” (S3)

About the “Do you have any suggestions for system improvement? describe” question, the users answered:

- “None.”(S1)
- “Something that would be interesting would be the possibility of browsing the internet, making use of the assistant. Older people have difficulties with new technologies, and sometimes browsing the internet is difficult. If the system allowed browsing the internet, I think it would be very useful.” (S2)
- “Be able to integrate with other devices, such as a smartwatch.” (S3)

## 6. Conclusions

Throughout this research, we show that the theme of ambient intelligence has been gaining attention in recent years. However, this theme has been a challenge in human-computer interactions since, currently, the interaction is no longer performed only through the graphical user interface, but through gestures or voice, for example. Furthermore, these environments require an advanced control system such as Homer, which is among the contributions of our work.

Homer, our approach, contains the following features: find objects, find a specific object, Identify when objects are out of the usual places, identify whether the environment light is on or off, and provide entertainment for solitude (i.e., free conversation, jokes, and inform the weather). Homer can be easily extended to include new features and technologies.

To validate our approach, we evaluated it with three HCI specialists. We understand that Homer respects usability, accessibility, natural interaction, features, and satisfaction criteria with these evaluations. However, improvements and new features were identified.

Among the contributions of this research, we highlight: the identification of the needs of older people in AAL and the proposition of an approach to ambient assisted living including an interactive multi-agent system to assist visually impaired and older people at home. Regarding the limitations of this work, we used a single camera in the environment. Thus, the information obtained in the object location feature had no depth. Therefore the information was not highly accurate, and we could not give information such as if one object was on top of another, or under another, for example. The use of Dialogflow can also be considered a limitation, as it was only possible for the user to make requests; the system could not talk to the user without receiving a request. Thus, it was not possible for the system, for example, to remind the user of something or, upon identifying a change, inform the user.

Furthermore, in our future work, we intend to (i) implement all features identified during this work that has not yet been implemented; (ii) make improvements identified by users, such as the voice configuration module; (iii) also, related to this, conduct training in cloud vision to identify more objects. We also intend to (iv) insert data privacy and security issues into Homer, (v) conduct user evaluations, and (vi) extend Homer for use with other chatbots (e.g., Rasa, Watson, Luis) to provide other image detection technologies (e.g., ROS, YOLOv3), and use more cameras and sensors.

## Figures and Tables

**Table 1:** HCI specialists profile.

Id	Age	Gender	Last Degree	Academic Formation	HCI Experience (Years)
S1	29–39	M	Postgraduate	Systems for internetand Ux Design	1–3
S2	29–39	M	MsC	Computer science	1–3
S3	40–50	M	Graduate	Information system	3–6

## Abbreviations

The following abbreviations are used in this manuscript:

AAL	Ambient Assisted Living
AmI	Ambient Intelligence
HCI	Human–Computer Interaction
MAS	Multi-Agent System
RFID	Radio Frequency Identification
SMS	Short Message Service
